# The 2017 Oslo conference report on neglected tropical diseases and emerging/re-emerging infectious diseases – focus on populations underserved

**DOI:** 10.1186/s40249-019-0550-8

**Published:** 2019-05-28

**Authors:** Katharina Klohe, John Amuasi, Joyce Moriku Kaducu, Ingeborg Haavardsson, Ekaterina Bogatyreva, Kristine Husøy Onarheim, Wendy Harrison, Frederik Kristensen, Clarissa Prazeres da Costa, Andrea S. Winkler

**Affiliations:** 1 Department of Neurology, Center for Global Health, Technical University of Munich, Munich, Germany; 2 Center for Global Health, Institute for Medical Microbiology, Immunology and Hygiene, Technical University of Munich, Munich, Germany; 3 African Research Network for Neglected Tropical Diseases (ARNTD), The Kumasi Centre for Collaborative Research in Tropical Medicine (KCCR), Kumasi, Ghana; 4 Ministry of Health, Kampala, Uganda; 5 Centre for Global Health, University of Oslo, Oslo, Norway; 6 Department of Global Public Health and Primary Care, University of Bergen, Bergen, Norway; 7 Schistosomiasis Control Initiative, Imperial College London, London, UK; 8 Coalition for Epidemic Preparedness Innovation, Oslo, Norway

**Keywords:** Neglected tropical diseases, Emerging infectious diseases, One health, SDGs, Poverty, Neglect

## Abstract

**Background:**

In 2017, the Centre for Global Health (CGH) at the University of Oslo in collaboration with the Coalition for Epidemic Preparedness Innovations (CEPI) and the Norwegian Agency for Development Cooperation (Norad) held a meeting to discuss together with leading figures in disease control, research and development the issue of neglected tropical diseases and emerging/re-emerging infectious diseases. This commentary has taken up this discussion and the conclusions drawn at this meeting to make a case for the opportunity the Sustainable Development Goals (SDGs) provide in highlighting the interconnectedness of factors that are relevant in the successful fight against neglected tropical diseases (NTDs) and emerging infectious diseases (EIDS).

**Main body:**

Despite NTDs being endemic and EIDS being epidemic, in order to prevent both disease groups effectively, it is important to appreciate that they share essential health determining factors, namely: neglect, poverty, a lack of access to clean water and sanitation facilities and an absence of or severely limited provision of healthcare as well as in many cases a zoonotic nature. Instead of looking to “simple disease management” for the answer, the SDGs help to understand the interplay of multiple priority areas and thereby help to promote a more holistic approach to addressing these two disease groups.

**Conclusions:**

Their commonalities mean that the Global Health community should leverage opportunities and efforts in the prevention and elimination of both NTDs and EIDs. Doing so using a One Health approach is considered to offer a “public health best-buy”. Concrete solutions are proposed.

**Electronic supplementary material:**

The online version of this article (10.1186/s40249-019-0550-8) contains supplementary material, which is available to authorized users.

## Multilingual abstracts

Please see Additional file 1 for translations of the abstract into the five official working languages of the United Nations.

## Background

In 2017, the Centre for Global Health (CGH) at the University of Oslo in collaboration with the Coalition for Epidemic Preparedness Innovations (CEPI) and the Norwegian Agency for Development Cooperation (Norad) held a meeting to discuss together with leading figures in disease control, research and development the issue of neglected tropical diseases (NTDs) and emerging / re-emerging infectious diseases (EIDs). This commentary intends to share this discussion and the conclusions drawn at the meeting. Underlying the discussions was a common understanding that much progress, in particular with regards to NTDs has already been made in recent years. Thus, with the aim to highlight leveraging opportunities in the prevention and management of NTDs and EIDs and the need to combine forces in a One Health manner, the authors will examine each disease area individually, before discussing the commonalities and the additional actions necessary to prevent and alleviate the associated burden.

## Main text

### Neglected tropical diseases

Neglected tropical diseases constitute a group of mainly communicable diseases (with snake bite envenomation being the first non-communicable disease that has been added to the list) that primarily affects communities in resource poor settings in tropical and subtropical conditions. From a focus on individual diseases such as Chagas disease and leishmaniasis, the term NTDs as a collective term for a number of diseases that were biologically dis-similar but affected the same neglected populations was coined in an attempt to raise awareness and funding for this group of diseases, considering that historically very little attention had been paid to them in comparison to the “big three” diseases, i.e. HIV/AIDS, malaria and tuberculosis (TB) [1]. Many consider NTDs to be a “chronic pandemic” due to the large number of people affected [2]. Arguably, since NTDs mainly concern people of lower socioeconomic status there is insufficient attention and financial resources from policy-makers and research efforts to develop new interventions. NTDs are highly prevalent both in remote rural areas, as well as in poor urban settings, mainly - but not exclusively - in low- and middle-income countries (LMICs) [3]. Importantly, NTDs are also increasingly prevalent in high income countries such as the United States of America and some southern European countries (e.g. Dengue fever, Chagas disease, leishmaniasis, opisthorchiasis and schistosomiasis) due to poverty, migration and climate change [4–6]. As previous LMICs have experienced large economic growth, the highest overall burden of NTDs is amongst poor people in large emerging-market economies in the G20 countries, such as India [3].

The World Health Organization’s (WHO) list of NTDs currently includes 20 diseases [7], while *PLoS Neglected Tropical Diseases* (PLoS NTD)– the only journal solely devoted to the research of NTDs – considers twice as many diseases to fit in this disease category [8]. A consolidated list of NTDs according to the WHO and PLoS NTD definitions is given in Table 1, indicating the nature of transmission as well as their reservoir. Overall, they constitute a diverse group of mainly infectious or communicable diseases with variable opportunities for elimination as a public health problem or control and a wide range of reservoirs from solely human to mainly zoonotic.

**Table 1:** Neglected tropical diseases: Overview of WHO and PLOS NTD lists, their main reservoir and mode of transmission

	Main reservoir			MODE of TRANSMISSION		
	Human	Zoonotic	Other^b^)	Oral/food-borne	Vector-borne	Other^c^)
Viral diseases						
Dengue and Chikungunya fevers^a^	X				X	
Japanese encephalitis		X			X	
Jungle yellow fever		X			X	
Other arboviral infections		X			X	
Rabies^a^		X				X^e^
Rift Valley fever		X			X	
Viral haemorrhagic fevers		X		X	X	X^d, e^
Bacterial diseases						
Bartonellosis	X	X			X	X^e^
Bovine tuberculosis in humans		X		X		
Buruli ulcer^a^		X^h^	X^h^			X^d^
Cholera			X	X		
Diarrhoeal diseases (Shigella, Salmonella, *E. coli*)	X			X		
Leprosy^a^	X	(X)				X^e^
Leptospirosis		X		X		
Trachoma^a^	X					X^e^
Treponematoses (Yaws, Endemic syphilis, Pinta)^a^	X					X^e^
Helminthic diseases						
Dracunculiasis^a^	X			X		
Echinococcosis^a^		X		X		
Food-borne trematodiases^a^		X		X		
Loiasis	X				X	
Lymphatic filariasis (LF)^a^	X				X	
Onchocerciasis^a^	X				X	
Schistosomiasis^a^	X	X				X^e^
Soil-transmitted helminthiases (Ascariasis, Hookworm disease, Trichuriasis, Strongyloidiasis)^a^	X			X		X^e^
Taenia solium (Neuro)Cysticercosis/Taeniosis^a^		X (only taeniosis)		X		
Toxocariasis and other larva Migrans diseases		X		X		X^e^
Protozoan diseases						
Amoebiasis	X			X		
Balantidiasis		X			X	
Chagas disease^a^		X			X	X^g^
Giardiasis	X			X		
Human African trypanosomiasis (HAT)^a^	X	X			X	
Leishmaniasis^a^	X	X			X	X^g^
Ectoparasitic diseases						
Myiasis		X			X	
Scabies^a^	X				X	
Snake bite envenoming			X			X^e^

Note: ^a^) indicates the 20 diseases categorised by WHO as belonging to the neglected tropical diseases; ectoparasitic and fungal infections as well as snakebite envenoming have recently been added in 2017 on the occasion of the 10th meeting of the Strategic and Technical Advisory Group for Neglected Tropical Diseases

^b^) Environment

^c^) unknown^d^, direct contact^e^, fetomaternal^g^

^h^) possible zoonotic and/or environmental reservoir

According to the Expanded Special Project for Elimination of Neglected Tropical Diseases, 1.5 billion people are affected by NTDs worldwide and it is estimated that between 350 000 and 500 000 die from them each year [9–13]. The Global Burden of Disease Study in 2016 attributed 18.79 million (26.06 million in 2010) DALYs (disability adjusted life years; calculated as the sum of the years of life lost due to premature mortality in the population and the years lost due to disability for people living with a health condition and its consequences) to those diseases categorised by WHO as belonging to the NTDs [14, 15]. In more in-depth calculations based on the 2010 Global Burden of Disease study an additional 21.84 million DALYs were attributed to diseases not listed by WHO as NTDs amounting to a total of 48 million DALYs (such in-depth calculations have not been performed with the 2016 Global Burden of Disease data) [16]. According to the G-Finder survey 2017 that looks at all the global funding spent on research and development on what it classifies as neglected not just neglected tropical diseases (USD 3203 million in 2016), 70% go to HIV/AIDS (81.55 million DALYs in 2010; 57.57 million, DALYs in 2016; funding of USD 1.10 billion, 2016), malaria (82.68 million DALYs in 2010; 56.20 million DALYs in 2016; funding of USD 576 million, 2016) and tuberculosis (49.40 million DALYs, 2010; 43.56 million DALYs, 2016; funding of USD 568 million, 2016) [17]. According to the G-Finder survey, the most poorly funded neglected diseases, i.e. leprosy, cryptococcal meningitis, Buruli ulcer, leptospirosis, trachoma and rheumatic fever in comparison each receive less than 0.5% of global funding [17, 18].

A concern in the management of NTDs is the focus that has been on the use and success of large-scale preventive chemotherapy (PC) by means of mass drug administration [19]. Medication that can be used this way is available for seven NTDs (lymphatic filariasis, onchocerciasis, schistosomiasis, soil-transmitted helminths, trachoma, yaws and scabies [strategies appear promising and require more research]). In the majority of cases it is donated by the pharmaceutical companies and represents the largest donation in history [19, 20]. Medications are distributed to large populations based on threshold mapping of at-risk population. Criticism has been raised at the reliance on a number of single medication, risking resistance development and a single mode of prevention for these diseases especially in the light of the lack of alternative drugs and the underfinanced developmental pipeline. In response to this, the NTD NGO Network has developed a framework that recognises the need for a more holistic approach to achieve sustainable progress, building on the five-pillar disease strategy outlined by WHO (PC, disease management, vector control, veterinary public health and water, sanitation and hygiene) [21]. This framework advocates for a more systematic approach to the inclusion of a broader range of interventions in NTD programme planning, considering environmental factors such as vector control, management of the animal reservoir in zoonotic diseases and engagement of the water, sanitation and hygiene sector as well as behaviour change and the need for social inclusion [22].

### Emerging and re-emerging infectious diseases

The grouping of diseases into emerging and re-emerging infectious diseases (EIDs) has been around for much longer compared to NTDs. WHO defines EIDs as those diseases “whose incidence in humans has increased during the last two decades or which threaten to increase in the near future. The term includes newly-appearing infectious diseases or those spreading to new geographical areas. It also refers to those that were easily controlled by chemotherapy and antibiotics but have developed antimicrobial resistance” [23].

Owing to recent attention to EIDs, development partners, WHO and public health agencies link the particular threat of how EIDs represent a health and security risk by crossing national borders as well as through climate change and urbanisation [13]. With our world becoming ever more interconnected and globalised, in particular via air travel as well as trade and migration routes, the risk of fast and uncontrolled spread of diseases across national and international borders, as well as continents, is increasing at a fast rate. Public health measures and health care systems that are breaking down as a result of conflict or economic collapse are also concerning risk factors for EIDs [24].

While much attention has been paid to viral infections, research suggests that 54% of EIDs are actually caused by bacteria, a large number of which reflect drug-resistant microbes [25]. The fact that WHO had to respond to 50 EID emergencies in 48 countries within six months around the end of 2017 and the beginning of 2018, nine of which were grade 3 emergencies, the highest level in WHO grading system, demonstrates how EIDs set the public health agenda [26]. These emergencies included cases of Middle East respiratory syndrome coronavirus (MERS-CoV) in Oman, Saudi Arabia, as well as the UK, the Plague in Madagascar, outbreaks of cholera in Kenya, Nigeria and Zambia and not to forget the second major outbreak and ongoing Ebola crisis in the Democratic Republic of the Congo, to name but a few [27, 28]. These outbreaks concern diseases that are on the WHO list of Blueprint priority diseases, meaning they pose a public health risk because of their significant epidemic potential and too little resources dedicated to countermeasures in the form of research and development (see also Table 2 for the complete WHO list of Blueprint diseases).

**Table 2:** WHO’s Blueprint priority diseases

	Zoonosis	Vector	Other
Crimean-Congo haemorrhagic fever (CCHF)	X	X	
Ebola virus disease and Marburg virus disease	X		
Disease X^a^	X	X	X
Lassa fever	X		
Middle East respiratory syndrome coronavirus (MERS-CoV) and severe acute respiratory syndrome (SARS)	X		
Nipah henipaviral diseases	X		
Rift Valley fever	X	X	
Zika virus	X	X	

Note: ^a^ “Disease X represents the knowledge that a serious international epidemic could be caused by a pathogen currently unknown to cause human disease, and so the R&D Blueprint explicitly seeks to enable cross-cutting R&D preparedness that is also relevant for an unknown “Disease X” as far as possible” (http://www.who.int/blueprint/priority-diseases/en/)

The significant burden EIDs can have on economies and public health including healthcare systems has become very apparent in the aftermath of the 2014 Ebola outbreak. The World Bank estimated the overall impact, covering the crisis in 2014 to 2015 as well as projections into 2016 on Guinea, Liberia and Sierra Leone at USD 2.8 billion in total. The healthcare systems were significantly affected by the Ebola outbreak, leading to a loss in healthcare workforce and reduced access to healthcare services, responsible for an additional 106 000 deaths in Guinea, Liberia and Sierra Leone, according to the Center for Disease Control and Prevention. These include 1091 additional deaths due to HIV/AIDS, 2714 additional deaths due to TB and 6818 additional deaths due to malaria [29].

Similar to NTDs, animals constitute an important reservoir of many pathogens causing EIDs. It is estimated that 60% of all human infectious diseases are of zoonotic origin and approximately 75% of all pathogens that lead to EIDs in humans are transmitted from the animal kingdom and in fact cause three out of five new diseases every year [30]. These percentages have been increasing over the last decades [25, 31, 32]. Rising population and subsequent increasing demand for land and food has led to farming land as well as village and city borders moving closer to wildlife’s natural habitat, increasing the chances for human-wildlife contact. Furthermore, animal transportation as part of global food chains not only propagates the spread of diseases amongst animals but also the risk for the spread to humans [26].

While the continued neglect of the chronic NTD pandemic primarily affect populations in resource poor settings, new epidemics of EIDs have gained attention due to their potential spread and risk to everyone, including those in high-income countries. EIDs have thus been recognized as posing a substantial threat to global health security. This has been particularly driven by the recent 2014 Ebola outbreak as well as the rising concerns regarding antimicrobial resistance. As a result, initiatives such as the Coalition for Epidemic Preparedness Innovation (CEPI), have emerged. CEPI is a coalition whose role is to finance and coordinate the development of new vaccines in order to prevent and or contain future infectious disease epidemics [33]. With initial funding of about USD 630 million CEPI has focused on developing vaccine candidates ready for large scale efficacy trials against three of WHO’s Blueprint priority diseases, namely Lassa fever, Middle East Respiratory Syndrome (MERS) and Nipah disease, all of which have large animal reservoirs (Table 2). In addition, CEPI focuses on helping finish the development of approved vaccines against Ebola [34]. Moreover, the coalition supports the development of well-characterized vaccine platforms to accelerate development of vaccines against unknown pathogens [35]. CEPI will also support innovations and effective processes for licensing and using vaccines, outcomes that may have impact beyond the specific diseases.

CEPI is an important initiative and Global Health player. The global health crisis of the 2014 Ebola outbreak has however illustrated that multiple reforms are essential in order to be adequately prepared for the next pandemic [36]. Moreover, many other EIDs still do not benefit from the needed attention and are still neglected in terms of funding, research and innovative measures for prevention, as well as healthcare system investments to meet the Sustainable Development Goals (SDGs) [37–39].

### NTDs, EIDs and the SDGs

As opposed to EIDs, many NTDs are of endemic nature but clearly an overlap exists such as in the case of leishmaniasis, which can cause outbreaks (see wars in Syria and Iraq) or *Helicobacter pylori*, which is classified as an EID but is endemic in nature (44% of the world is colonized) [40]. Nevertheless, for the prevention of both disease groups, it is important to appreciate that it is the most underserved and most socio-economically disadvantaged population groups that are most affected and at risk. In addition, these community often live in areas where environmental and ecological factors also interact to promote the spread of diseases [25, 41, 42], for example by living in close proximity to animals – domestic and wild - and experience a lack of access to clean water and sanitation facilities [4]. Absence of or severe deficits in healthcare systems or the inability to access these healthcare services also compound the impact of these diseases.

**Fig. 1:**
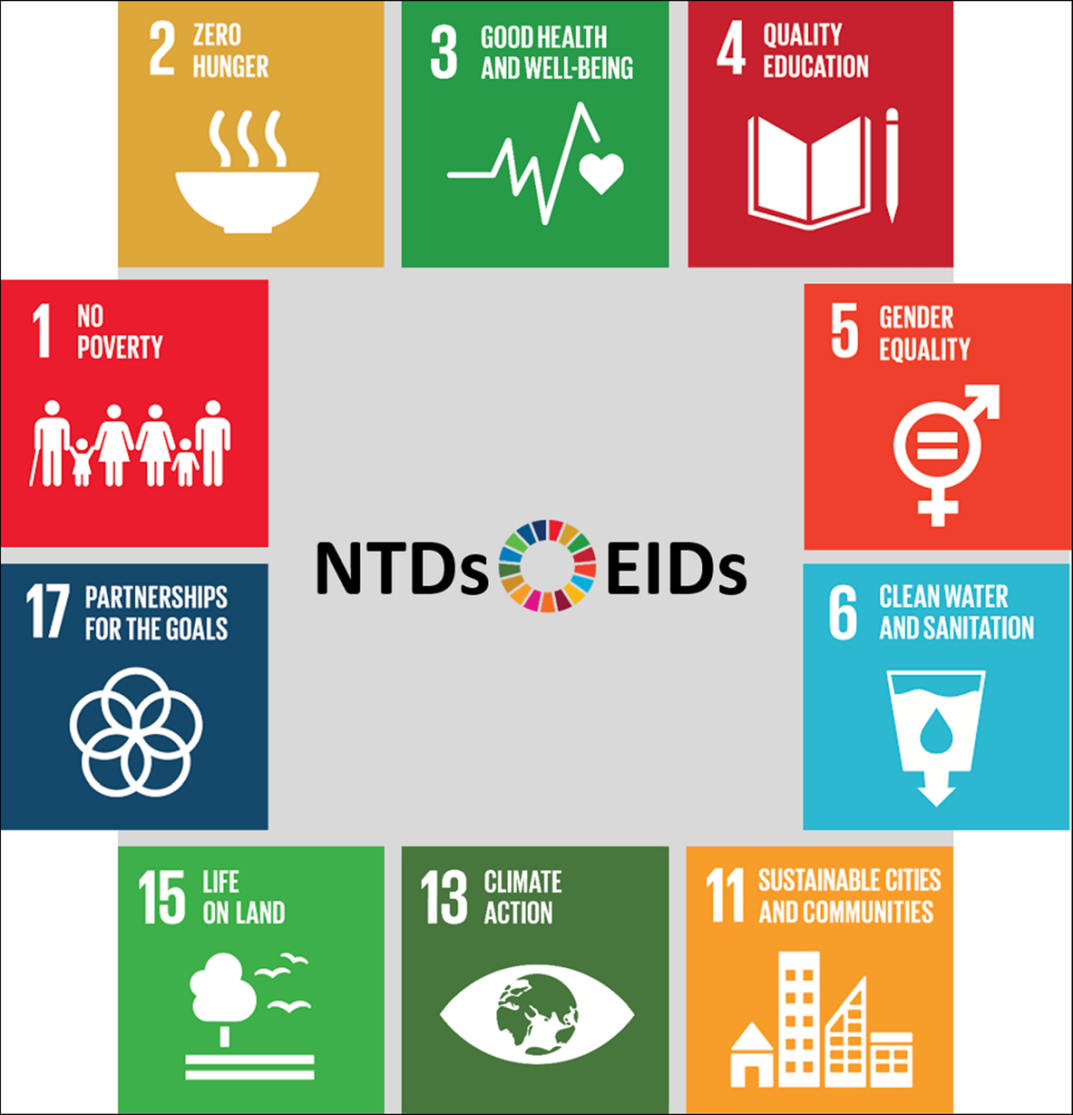
The SDGs central to addressing NTDs and EIDs in a multidisciplinary way ©Center for Global Health, Technical University of Munich

It hence becomes very apparent that effectively dealing with these disease groups is not a matter of “simple disease management”, but that integrated and interdisciplinary approaches are required to improve health sustainably. The 2030 Sustainable Development Goals Agenda offers a great opportunity as it helps to highlight priority areas for action and promotes a more holistic approach [43]. Figure 1 below highlights those SDGs central to addressing both NTDs and EIDs effectively.

Particularly relevant to NTDs and EIDs is the connection between the health of the environment, the health of the people, as well as the health of animals. The latter is not explicitly stated or referred to in the SDGs but is of equal importance. We refer to this as One Health. One Health is understood as “an approach to designing and implementing programmes, policies, legislation and research in which multiple sectors communicate and work together to achieve better public health outcomes” [44]. We therefore urge the global community to continue to advocate for integrated cross-sectorial approaches in the prevention and management of diseases that predominantly affect the poor and neglected people and that activities addressing NTDs and EIDs take an approach that considers human, animal and environmental health conjointly. This also incorporates the control of vectors involved in the transmission of diseases (see Table 1) as well as the prevention of their spread to and risk for humans.

### One health and global politics

At the second annual review of WHO Blueprint diseases in 2018 (Table 2), the impact of environmental issues on the Blueprint diseases was set on the agenda for upcoming reviews [34]. This demonstrates two important aspects: Firstly, as we have stated above, EIDs, similarly to NTDs, require a One Health approach, which includes environmental issues if diseases are to be meaningfully and sustainably prevented and managed. Secondly, a One Health, interdisciplinary approach is not yet sufficiently driving activities to prevent such diseases from emerging.

In a speech in February 2018 in Thailand on the threat of EIDs WHO’s Director General, Dr. Tedros Adhanom Ghebreyesus said, “We are setting ourselves a goal that over the next five years, 1 billion more people will be better protected from epidemics and other health emergencies” [26]. The corresponding WHO’s Thirteenth General Programme of Work, 2019–2023, emphasises four points WHO also seeks to ensure: 1) access to essential life-saving health services and public health interventions, 2) appropriate equipment of countries to mitigate risks from high-threat infectious hazards, 3) that any gaps are identified in health emergency preparedness (under the International Health Regulations) by countries themselves, and 4) that national health-emergency programmes receive support by WHO Health Emergencies Programme [45].

These four points are reiterated by Kluge and colleagues in their article on Global Health security and the International Health Regulations requirements, in which they go even further making a very valid case for the necessity of joining up “thinking between health system strengthening activities and health security efforts for prevention, alert and response” [46]. The authors also acknowledge the crucial role of the veterinary public health and agricultural sectors in responding to the potential threats associated with the rearing and moving of animals and livestock. However, we argue that more urgent action and financial commitments are needed to promote closer collaboration and equal contribution from the animal and environmental sectors. Health systems alone cannot address socio-economic determinants of health such as water, sanitation, and hygiene, proximity of living with animals, animal health overall and the need of surveillance of livestock and where possible wildlife as well as vectors. Further, poverty is a crosscutting issue. The tendency of people living in resource-poor settings to fall ill and endure the harmful economic effects of seeking healthcare, as well as underfunded health systems demonstrate how poverty is a chronic threat for current and future epidemics and pandemics. Investment in the health of the poor, poverty diseases and universal health coverage is a necessity to reach the SDGs on health.

That this is possible and effective was shown by Joyce Moriku Kaducu, the Minister of State for Primary Health Care in Uganda who spoke at the meeting in Oslo about the progress and challenges with regards to the NTDs and was able to highlight key factors that have contributed to the Ugandan success, such as the elimination of the Guinea worm (Uganda was certified free of Guinea worm in 2009) and the reduction in the burden of disease for many NTDs. Progress has also been made, for example, with regards to trachoma, with a reduction of people at risk from an estimated 10 million in 2014 to 300 000 in 2018 [47]. Amongst those key factors were 1) collaborating with other ministries such as agriculture, animal industries and fisheries and education, 2) interventions that were pro-poor and targeted communities living in slums and poor rural and urban areas as well as, 3) women and children were empowered both with knowledge and economically about NTDs associated with poor sanitation since they are typically the most affected population group. Thus, paying attention to the underserved and working in partnership with other disciplines and ministries as well as empowering women is effective and it points to the importance of having the attention of the politicians [48]. Despite the successes, it is important to note that cross-border transmission between the Democratic Republic of the Congo and South Sudan remains a challenge for elimination of NTDs as a public health problem in endemic districts in Uganda.

### “A public health best buy”

The meeting in Oslo highlighted the need and interest of the Global Health community to leverage opportunities in the prevention and elimination of both NTDs and EIDs. Delivering on NTDs and EIDs through both cross-sectoral and targeted initiatives will be beneficial to both human and animal health and thus contribute positively to overall health and economic security. Schar and colleagues even speak of an estimated global benefit of over USD 30 billion annually as a result of “a yearly investment of USD 1.9–3.4 billion to strengthen animal and human health systems” [32]. According to the World Bank Report that forms the basis of these estimated, investments need to be directed amongst others to staffing and capacity of veterinary professionals, surveillance and control mechanisms and general management and regulations in low and middle income countries [49]. In an important next step, veterinary and human health systems need to communicate and share data in order to identify potential areas of risk and gaps in control mechanisms. The extent of the benefit can be explained by the fact that severe economic damages associated with pandemics, such as seen in the 2014 Ebola crisis, as well as estimated for NTD infections, would be avoided. The public health best buy thus means that relatively small investments that are wisely allocated in order to leverage on existing systems, improve surveillance, prevent chronic morbidities of disease and link human, animal and environmental health can help to achieve large-scale (and if maintained long-term) health and economic improvements as well as health security [50].

Despite the promising economic case that can be made, panellists in Oslo wondered whether the global landscape with its current activities favour drug developments and donations rather than effectively using treatment and prevention strategies that already exist and scaling them up to everyone in need. Combating NTDs and EIDs in many cases can be effective at primary healthcare and district healthcare facilities. Dr. Mwelecele Malecela, Director WHO Department of Control of Neglected Tropical Diseases, has just made such a case at the global conference on mycetoma in Sudan, February 2019, stating that, “An essential first step could be to look at how we can integrate mycetoma interventions within primary health care delivery*”* [51]. Therefore, we think it necessary for coordinated funding and capacity building to also be directed towards strengthened primary health care services for sustainable impact to be achieved. This would imply active involvement of politicians and governments and investment in universal health coverage [39]. Yet, as was argued by the panellists in Oslo, while neglect is a political issue (neglect of the underserved people that are particularly affected by both NTDs and EIDs), technical discussions or philanthropic efforts, though absolutely necessary and valuable, may divert our attention from the role of politicians, governments and responsible decision-makers [42]. Unless this neglect is addressed, the discussants worried that the current scenario of resource constraints faced by people and animals will be maintained [25, 52].

### Oslo proceedings

It was concluded in Oslo that the burden of NTDs and EIDs disproportionally affect the most neglected and underserved populations, in LMICs. They are diseases of poverty, neglected people, neglected research and development, knowledge, data and access to diagnostic, treatment and prevention mechanisms. Furthermore, this neglect takes place at the local, national and global level. Strengthening health systems, community ownership of disease surveillance and programmes as well as different and/or novel approaches to dealing with and preventing NTDs and EIDs simultaneously i.e. addressing living conditions, behaviour change and disease education, are required to combat the current and future challenges. Reciprocally, integrating programmes into health systems that effectively combat NTDs and prevent the spread of EIDs has the potential to accelerate progress towards Universal Health Coverage while advancing the broader SDGs for 2030. Finally, it was considered imperative to also address the zoonotic roots of NTDs and EIDs, the majority of which are not prioritised and targeted by pharmaceutical companies and governments. Addressing zoonotic diseases by taking a One Health approach is a great opportunity to tackle NTDs and EIDs in a holistic and multidisciplinary way [42, 53]. This has now also been taken up by a Lancet One Health Commission of which some of the authors of the current paper are part and that will hold its first meeting again in Oslo at the beginning of May 2019.

The key challenges and solutions that were highlighted can be found in the summary box below.

Summary Box: Key challenges and solutions for combatting NTDs and EIDs discussed at the Oslo conference

- Lack of incentives for R&D of vaccines and treatments for both NTDs and EIDs.
- Reaching neglected populations.
- Building resilient health systems.
- Timely access to already-existing treatments in rural and urban clinics in the Global South.
- Including engaged communities, civil society, political stakeholders.
- Amplifying human and financial resources from both local and international sources.
- Strengthening disease surveillance and reporting.
- Implementing infection control measures.
- Prioritizing a disease for NTD lists.
- Joining forces in global health research and building common frameworks based on a common set of principles.
- Calling for One Health research activities and strategies.
- Research to anticipate future climate patterns and natural disasters, which affect agriculture and livelihoods of people in NTD and EID affected regions and thus pre-empting the spread of NTDs and EIDs.
- Addressing the SDGs, which run through the heart of the problem of poverty, sanitation, education, hunger, health of the planet, health of cities and health of the people.

## Conclusions

In our view, a multidisciplinary One Health approach, which requires collaboration across sectors and ministries, is indispensable for successful prevention, control and elimination of most NTDs and EIDs and should thus be given highest priority. This will yield gains for patients affected by NTDs and EIDS, but also other causes of premature illness and deaths. The following solutions were proposed at the meeting in Oslo and are emphasised again in this commentary: 1) pushing more forcefully for a One Health approach that includes advocacy for and investment in animal health research and community livelihood that then become core components of national programmes, 2) encouraging and supporting countries to develop national action plans (NAPs) that address both, the prevention of NTDs and EIDs by considering to leverage opportunities in terms of for example the strengthening of human and animal health systems and their coordination, 3) fostering research to anticipate future climate patterns, natural disasters, which affect agriculture and livelihoods of people in affected regions and thus pre-empting the spread of neglected- and emergence of new- diseases, 4) strengthening disease surveillance and reporting, 5) continued development of medical countermeasures against NTDs and EIDs, and finally 6) addressing the SDGs, which run through the heart of the problem of poverty, sanitation, education, hunger, and health of the people. All these require neglected populations to be reached. Joining forces in global health research and policy are considered one of the most essential building blocks for all of the above. This discussion will be taken further in the upcoming One Health Lancet Commission, co-chaired by this article’s co-authors Dr. John Amuasi and Prof Andrea Winkler.

## Additional file

Additional file 1: Multilingual abstracts in the five official working languages of the United Nations. (PDF 568 kb)

## Abbreviations

CEPI: Coalition for Epidemic Preparedness Innovation
EIDs: Emerging/re-emerging infectious diseases
LMICs: Low and middle income countries
NTDs: Neglected tropical diseases
SDGs: Sustainable Development Goals
TB: Tuberculosis
WHO: World Health Organization
