# Supplementary material for: The 2017 Oslo conference report on neglected tropical diseases and emerging/re-emerging infectious diseases – focus on populations underserved
Source: Infect Dis Poverty. 2019 May 28;8:40. doi: 10.1186/s40249-019-0550-8 (PMC6537383; doi:10.1186/s40249-019-0550-8)
Supplement: Supplementary file 1 — Multilingual abstracts in the five official working languages of the United Nations. (PDF 568 kb) [file 40249_2019_550_MOESM1_ESM.pdf]

Translation of the abstract into the five official working languages of the United Nations

**التركيز على السكان المحرومين**

آنا كاثرينا كلوه ، جون أمواس ، جويس موريكو كادوكو ، إنجيبورج هافاردسون ، إيكاترينا بوغاتريفا ، كريستين هوسوي أونارهيم ، ويندي هاريسون ، فريدريك كريستنسن ، كلاريسا برايزريس دا كوستا ، أندريا إس وينكلر

خلفية علمية
في عام 2017 ، عقد مركز الصحة العالمية (CGH) في جامعة أوسلو بالتعاون مع التحالف من أجل ابتكارات التأهب للوباء (CEPI) والوكالة النرويجية للتعاون الإنمائي (Norad) اجتماعًا لمناقشة الشخصيات البارزة في مكافحة الأمراض والبحث والتطوير في قضية الأمراض المدارية المهملة والأمراض المعدية الناشئة / الناشئة
تناول هذا التعليق هذه المناقشة والاستنتاجات التي تم التوصل إليها في هذا الاجتماع لإثبات وجود الفرصة التي توفرها أهداف التنمية المستدامة (SDGs) في إبراز الترابط بين العوامل ذات الصلة في المعركة الناجحة ضد NTDs و EIDs.
الموضوع الرئيسي
على الرغم من أن الأمراض المدارية المهملة (NTDs) مستوطنة وأن الأمراض المعدية الناشئة (EIDS) وبائية ،و من أجل منع كلتا المجموعتين من الأمراض بشكل فعال ، فمن المهم أن نقدر شراكتهما في العوامل المحددة الصحية الأساسية ، وهي: الإهمال ، الفقر ، نقص الحصول على المياه النظيفة ومرافق الصرف الصحي وغياب أو توفير الرعاية الصحية المحدودة للغاية وكذلك في حالات كثيرة أن هذه الأمراض حيوانية المصدر.
بدلاً من البحث عن "إدارة بسيطة للمرض" للحصول على الجواب ، تساعد أهداف التنمية المستدامة على فهم التفاعل بين مجالات متعددة ذات أولوية ، وبالتالي تساعد على تعزيز نهج أكثر شمولية لمعالجة هاتين المجموعتين المرضيتين
استنتاج
تعني القواسم المشتركة بين الأمراض المدارية المهملة والأمراض المعدية الناشئة / أنه ينبغي لمجتمع الصحة العالمية الاستفادة من الفرص والجهود المبذولة في مجال الوقاية والقضاء على كل هذه الأمراض والقيام بذلك باستخدام نهج One Health. يعتبر بمثابة "الشراءالأفضل لالصحة العامة". يتم اقتراح حلول ملموسة.
لكلمات الدالة
الأمراض المدارية المهملة ، الأمراض المعدية الناشئة ، صحة واحدة ، أهداف التنمية المستدامة ، الفقر ، الإهمال

Translated from English version into Arabic by Prof Nagwar Elkhafif

**关于被忽视热带病和新发/再发传染病的 2017 年奥斯陆会议报告-专注于医疗条件薄弱人群**

Anna Katharina Klohe, John Amuasi, Joyce Moriku Kaducu, Ingeborg Haavardsson, Ekaterina Bogatyreva, Kristine Husøy Onarheim, Wendy Harrison, Frederik Kristensen, Clarissa Prazeres da Costa, Andrea S. Winkler

**摘要**

**引言：** 2017 年，奥斯陆大学全球卫生中心（CGH）与流行病应对创新联盟（CEPI）和挪威发展合作机构（Norad）共同召开会议，讨论疾病防治，被忽视的热带病和新发/再发传染病的相关研发问题。本文汇总了此次会议的讨论事项和结论，旨在为可持续发展目标（SDG）中强调的成功对抗被忽视热带病（NTDs）和新发传染病（EIDS）相关因素的关联性提供参考。
**主要内容：**尽管 NTDs 是地方流行，EIDs 正在蔓延，为了有效预防这两类疾病，重要的是要认识到它们共享的影响健康的决定性因素，如：被忽视、贫困、清洁用水和卫生设施的缺乏、医疗保健服务的缺失或严重不足、在很多情况下是否为人兽共患病。可持续发展目标不是寻求“简单的疾病管理”，而是帮助理解多个优先领域的相互作用，进而以更全面的方法来防治这两类疾病。
**结论：**被忽视热带病和新发传染病的共同点意味着，全球卫生界应该抓住机会，努力预防和消除这两类疾病，可以采用同一健康的方法提供“公共健康最佳服务”，具体的方案措施已提上日程。

Translated from English version into Chinese by Fan Yang, edited by Pin Yang

CDC CHINESE CENTER FOR DISEASE CONTROL AND PREVENTION
NATIONAL INSTITUTE OF PARASITIC DISEASES

**Compte-rendu de la Conférence d’Oslo 2017 sur les maladies tropicales négligées et les maladies infectieuses émergentes ou réémergentes - Gros plan sur les populations sous-desservies**

Anna Katharina Klohe, John Amuasi, Joyce Moriku Kaducu, Ingeborg Haavardsson, Ekaterina Bogatyreva, Kristine Husøy Onarheim, Wendy Harrison, Frederik Kristensen, Clarissa Prazeres da Costa, Andrea S. Winkler

**Résumé**

**Contexte:** En 2017, le Centre pour la santé mondiale (CGH) de l’Université d’Oslo, en collaboration avec la Coalition pour les innovations en matière de préparation aux épidémies (CEPI) et l’Agence norvégienne pour la

coopération au développement (Norad), a organisé une réunion pour débattre de la question des maladies tropicales négligées et des maladies infectieuses (ré)émergentes avec des spécialistes éminents de la lutte contre les maladies, de la recherche et du développement. Le présent commentaire rend compte de cette discussion et des conclusions qui en ont été tirées afin de démontrer l’opportunité qu’offrent les objectifs de développement durable (ODD) en mettant en lumière l’interdépendance des facteurs déterminants du succès de la lutte contre les maladies tropicales négligées (MTN) et les maladies infectieuses émergentes (MIE).
**Discussion:** Bien que les MTN soient endémiques et les MIE épidémiques, on ne peut prévenir efficacement ces deux groupes de maladies qu’en comprenant bien qu’ils ont en commun certains déterminants de la santé essentiels: négligence, pauvreté, manque d’accès à l’eau potable et à des installations sanitaires et absence ou disponibilité très restreinte de soins de santé, auxquels s’ajoutent, dans de nombreux cas, un caractère zoonotique. Au lieu de rechercher comme solution une « gestion simple de la maladie », les objectifs de développement durable aident à comprendre les interactions entre de multiples domaines prioritaires et contribuent ainsi à promouvoir une approche plus globale de la gestion de ces deux groupes de maladies.
**Conclusions:** Les points communs entre les MTN et les MIE créent des opportunités d’agir et des approches que la communauté mondiale de la santé doit mettre à profit pour la prévention et l’élimination des maladies appartenant à ces deux groupes. On considère que ces efforts appuyés sur une approche « One Health » représentent un « mieux-disant pour la santé publique ». Des solutions concrètes sont proposées.

Translated from English version into French by Amélie Gärtner, proofread by Suzanne Assenat, through

TRANSLATORS
WITHOUT BORDERS

**Отчет конференции в Осло 2017 года по забытым тропическим болезням и новым/вновь возникающим инфекционным болезням - в фокусе необеспеченное население**

Анна Катарина Клохе, Джон Амуаси, Джойс Морику Кадуку, Ингеборг Хаавардссон, Екатерина Богатырева, Кристина Хусей Онархайм, Венди Харрисон, Фредерик Кристенсен, Кларисса Празерес да Коста, Андреа С. Винклер

**Аннотация**

**Предпосылки:** В 2017 году Центр глобального здравоохранения (CGH) в Университете Осло в сотрудничестве с Коалицией за инновации в области обеспечения готовности к эпидемиям (CEPI) и Норвежским агентством по сотрудничеству в целях развития (Norad) провели встречу для обсуждения с ведущими специалистами по контролю за болезнями, исследованиям и развитию проблемы забытых тропических болезней и возникающих/вновь возникающих инфекционных заболеваний. В настоящем комментарии эта дискуссия и выводы, сделанные при обсуждении вопросов на ней, взяты в контексте тех возможностей, которые цели устойчивого развития дают при выяснении взаимосвязи факторов, относящихся к успешной борьбе с забытыми тропическими болезнями (НТД) и новыми возникающими инфекционными заболеваниями (EIDS).
**Основная часть:** Несмотря на то, что NTD являются эндемичными, а EIDS эпидемическими, для эффективного предотвращения обеих групп заболеваний важно понимать, что их объединяют общие определяющие здоровье факторы, а именно: заброшенность, бедность, отсутствие доступа к чистой воде и санитарно-техническим средствам и отсутствие или крайне ограниченное медицинское обслуживание, а также, во многих случаях, зоонозный характер. Вместо того, чтобы искать ответ «просто работая с болезнями», ЦУР помогают понять взаимодействие нескольких приоритетных областей и тем самым помогают продвигать более целостный подход в отношении этих двух групп болезней.
**Выводы:** Общие черты таких болезней показывают, что сообщество глобального здравоохранения должно использовать возможности и усилия для предотвращения и ликвидации как NTD, так и EID. Предполагается, что использование подхода One Health обеспечивает «лучший вариант общественного здравоохранения». Предлагаются конкретные решения.

Translated from English version into Russian by Kenul Aliyeva-Aqarzayeva, proofread by Alexander Somin, through

TRANSLATORS
WITHOUT BORDERS

**Informe de la Conferencia de Oslo 2017 sobre enfermedades tropicales desatendidas y enfermedades infecciosas emergentes y reemergentes - Enfoque en las poblaciones marginadas**

Anna Katharina Klohe, John Amuasi, Joyce Moriku Kaducu, Ingeborg Haavardsson, Ekaterina Bogatyreva, Kristine Husøy Onarheim, Wendy Harrison, Frederik Kristensen, Clarissa Prazeres da Costa, Andrea S. Winkler

**Resumen**

**Antecedentes:** En 2017, el Centro para la Salud Global (CGH) de la Universidad de Oslo, en colaboración con la Coalición para las Innovaciones en Preparación para las Epidemias (CEPI) y la Agencia Noruega de Cooperación para el Desarrollo (Norad), celebraron una reunión junto con figuras destacadas en el desarrollo, la investigación y el control de enfermedades para debatir el problema de las enfermedades tropicales desatendidas y las enfermedades infecciosas emergentes y reemergentes. Este comentario ha retomado esta discusión y las conclusiones extraídas de esta reunión para defender la oportunidad que brindan los Objetivos de Desarrollo Sostenible (ODS) al resaltar la interconexión de factores que son relevantes en la lucha exitosa contra las enfermedades tropicales desatendidas (ETD) y las enfermedades infecciosas emergentes (EIE).

**Parte principal:** A pesar de que las ETD son endémicas y las EIE son epidémicas, para prevenir ambos grupos de enfermedades de manera efectiva, es importante apreciar que comparten factores determinantes de salud esenciales, a saber: abandono, pobreza, falta de acceso a instalaciones de agua potable y saneamiento y ausencia de prestación de asistencia sanitaria, así como también en muchos casos naturaleza zoonótica. En lugar de buscar como respuesta una "gestión simple de la enfermedad", los ODS ayudan a comprender la interacción de múltiples áreas prioritarias y, por lo tanto, ayudan a promover un enfoque más integral para abordar estos dos grupos de enfermedades.

**Conclusiones:** Sus puntos en común significan que la comunidad del Centro para la Salud Global debe aprovechar las oportunidades y los esfuerzos en la prevención y en la eliminación tanto de las ETD como de las EIE. Se considera que el uso de un enfoque de One Health ofrece una "mejor opción de salud pública". Se proponen soluciones concretas.

Translated from English version into Spanish by Patricia Cassoni, proofread by Ivana Burges, through

TRANSLATORS WITHOUT BORDERS
